# Prevalence, Molecular Epidemiology, and Clinical Characteristics of Human Bocavirus Among Patients with Acute Gastroenteritis in Northern Brazil During 2017–2022

**DOI:** 10.3390/v17010079

**Published:** 2025-01-09

**Authors:** Yasmim Gabrielly Souza Sousa, Carolina Alcântara Maneschy, Carolina Costa Monteiro, João Victor Souza Rodrigues, Patrícia Santos Lobo, Dielle Monteiro Teixeira, Jones Anderson Monteiro Siqueira, Kenny Costa Pinheiro, Hugo Reis Resque, Luciana Damascena Silva, Sylvia Fátima dos Santos Guerra, Luana Silva Soares

**Affiliations:** 1 Postgraduate Program in Virology, Teaching and Scientific Information Section, Evandro Chagas Institute, Secretariat of Health Surveillance and Environment, Brazilian Ministry of Health, Ananindeua 67.030-000, Pará, Brazil; yasmim.g.s.sousa@gmail.com (Y.G.S.S.); carolmaneschy19@gmail.com (C.A.M.); 2 Laboratory of Gastroenteric Virus, Virology Section, Evandro Chagas Institute, Secretariat of Health Surveillance and Environment, Brazilian Ministry of Health, Ananindeua 67.030-000, Pará, Brazil; carolinamonteiro1914@hotmail.com (C.C.M.); rpgjjoao70@gmail.com (J.V.S.R.); patricialobo@iec.gov.br (P.S.L.); dielleteixeira@iec.gov.br (D.M.T.); jonessiqueira@iec.gov.br (J.A.M.S.); kennybiotec@gmail.com (K.C.P.); hugoresque@iec.gov.br (H.R.R.); lucianasilva@iec.gov.br (L.D.S.); sylviaguerra@iec.gov.br (S.F.d.S.G.)

**Keywords:** human bocavirus, acute gastroenteritis, viral coinfection, genotypes

## Abstract

Acute gastroenteritis (AG) is a major illness in early childhood. Recent studies suggest a potential association between human bocavirus (HBoV) and AG. HBoV, a non-enveloped virus with a single-strand DNA genome, belongs to the Parvoviridae family. This study aimed to describe the frequency of HBoV in Northern Brazil using samples from patients with AG collected between 2017 and 2022. Fecal samples obtained from the viral gastroenteritis surveillance network at the Evandro Chagas Institute (IEC) were analyzed. Fecal suspensions (20%) were prepared, and the viral genome was extracted. PCR and nested-PCR were employed to detect HBoV, followed by nucleotide sequencing to identify viral types. Out of 692 samples, HBoV positivity was detected in 9.2% of cases (64/692). Genotypes HBoV-1, HBoV-2, HBoV-3, and HBoV-4 were found in 42.5% (17/40), 22.5% (9/40), 32.5% (13/40), and 2.5% (1/40) of the specimens, respectively. Co-infections with HBoV and other enteric viruses occurred in 48.3% (31/64) of cases, with RVA being the most frequent (31.2%, 20/64). The study results underscore the importance of continuous monitoring and further research to better understand the seasonality, coinfection patterns, and genetic variability of HBoV.

## 1. Introduction

Globally, acute gastroenteritis (AG) is a clinical condition that represents one of the main morbidity and mortality causes in children under five years old [1]. Annually, AG is responsible for 443,832 deaths among children under 5 years old, and 50,851 deaths among children between 5 and 9 years old [2]. It is characterized by inflammation of the gastrointestinal tract, resulting in symptoms such as diarrhea, vomiting, and abdominal pain [3]. *Rotavirus* group A (RVA), norovirus (NoV), astrovirus (HAst), sapovirus (SaV), and enteric adenovirus (types 40/41) (HAdV) are the pathogens most associated with this condition. However, human bocavirus (HBoV) has emerged as a potential pathogen associated with AG [4].

HBoV is a non-enveloped icosahedral virus that belongs to the *Parvoviridae* family and *Parvovirinae* subfamily, and is a *Bocaparvovirus* genus. It consists of a single-stranded DNA (ssDNA) genome of 4.7–5.7 kilobases that encodes five nonstructural proteins (NS1 to NS4 and NP1) and three structural capsid proteins (VP1, VP2, and VP3) based on the HBoV-1 structure [5]. HBoV was first described in 2005 and initially linked to respiratory infections [6], but subsequent research also identified its presence in fecal samples, suggesting a possible association with AG [7,8]. In Brazil, the first HBoV detection in fecal samples occurred in 2007 [9]. Since then, many studies have been conducted to understand its prevalence and impact on human health [10,11].

HBoV is classified into four distinct species that can affect humans: HBoV-1, HBoV-2, HBoV-3, and HBoV-4. HBoV-1 is closely related to respiratory diseases, as several reports have linked it to asthma, colds, pneumonia, and other respiratory diseases [12,13]. On the other hand, HBoV-2, HBoV-3, and HBoV-4 species are commonly detected in AG cases but rarely found in respiratory samples [14,15,16].

Although the relationship between HBoV and AG is not well established, this agent has been frequently detected in stool samples from patients with diarrhea symptoms, especially in children aged between 6 and 24 months. Worldwide, Guido et al. (2016) [17] estimated that there is an HBoV total prevalence of 5.9% in gastrointestinal infections, and HBoV frequency in fecal samples ranged from 1.3% to 63%, depending on the geographical region. Thus, it is suggested that this virus may be an etiological agent of AG [9,15,16,18].

In this context, it is essential to understand the role of HBoV in AG for a better understanding of this agent as a causative factor of the disease and its circulation pattern in the population. This study aimed to evaluate the prevalence, clinical features, and genotype distribution of HBoV in seven states in the North Region of Brazil, using samples collected from patients with AG between 2017 and 2022.

## 2. Materials and Methods

### 2.1. Clinical Specimens and Ethical Aspects

For this cross-sectional study, 692 fecal samples from child and adult patients from the North Region of Brazil with AG symptoms (diarrhea, vomiting, and fever lasting 7 days) were collected between January 2017 and December 2022. In the present analysis, the definition of AG was 3 or more loose or liquid stools per day (or more frequent passage than is normal for the individual) or vomiting (≥1 episode in 24 h). Fecal samples were collected from sentinel sites at central laboratories in seven Brazilian states and sent to Evandro Chagas Institute, a National Rotavirus Reference Laboratory of the Brazilian Ministry of Health; the samples had already been previously tested for RVA and NoV.

### 2.2. Nucleic Acid Extraction

Viral nucleic acids (DNA and RNA viruses) were extracted from 20% fecal suspensions with Tris–calcium buffer (pH = 7.2) (Sigma-Aldrich, St. Louis, MO, USA) using the isothiocyanate/silica method [19]. The isolated nucleic acid was kept frozen at −70 °C until the molecular analysis was carried out. In each extraction procedure, RNAse/DNAse-free water (Gibco, Loughborough, UK) was used as a negative control.

### 2.3. HBoV Molecular Detection

HBoV detection was performed via PCR, followed by nested PCR, using two sets of primers that targeted a variable VP1/VP2 region, as described previously [4]. The PCR products were detected using electrophoresis on 1.5% agarose gel. The presence of HBoV was determined through a specific-sized amplicon corresponding to the second round of nested PCR of 576 bp, after being stained with a nucleic acid staining solution and visualized with a UV transilluminator (Vilber Loumart, Munich, Deutschland).

### 2.4. Molecular Characterization and Phylogenetic Analysis

The amplicon products were purified using a QIAquick PCR Purification Kit (QIAGEN, Valencia, CA, USA). Sequencing reactions were performed with the same primers from the nested-PCR using a BigDye^TM^ Terminator v. 3.1 Cycle Sequencing Kit (Applied Biosystems, Foster City, CA, USA) [20]. Subsequently, the reactions were purified and then run on an ABI Prism 3130xl genetic analyzer (Applied Biosystems, Foster City, CA, USA).

The obtained sequences were aligned and edited using Geneious Prime software (v.7) (Biomatters Ltd., Auckland, New Zealand) [21]. The sequence alignment, together with other related sequences available on GenBank (www.ncbi.nlm.nih.gov, accessed on 30 June 2024), was performed using the Basic Local Alignment Search Tool (BLAST) to confirm the genotypes in terms of closest homology sequence. The phylogenetic trees of the HBoV partial VP1/VP2 genes were constructed using the maximum likelihood method using FastTree software (v.2.1.11), including the GTR + Gamma + F nucleotide substitution model. Bootstrap values of the nodes indicated the support of 1000 replicas, obtaining reproducible results and providing the clusters with greater reliability. Partial nucleotide sequences from this study were deposited in the NCBI GenBank database under the access numbers PQ553014-PQ553053.

### 2.5. Statistical Analysis

Statistical analyses were performed using Jamovi Software (The Jamovi Project, 2024. Version 2.5). Bivariate analysis was carried out to verify the association between independent variables and HBoV positivity using the chi-square test (x^2^). A *p*-value ≤ 0.05 was considered statistically significant.

## 3. Results

### 3.1. HBoV Detection

**Figure 1:**
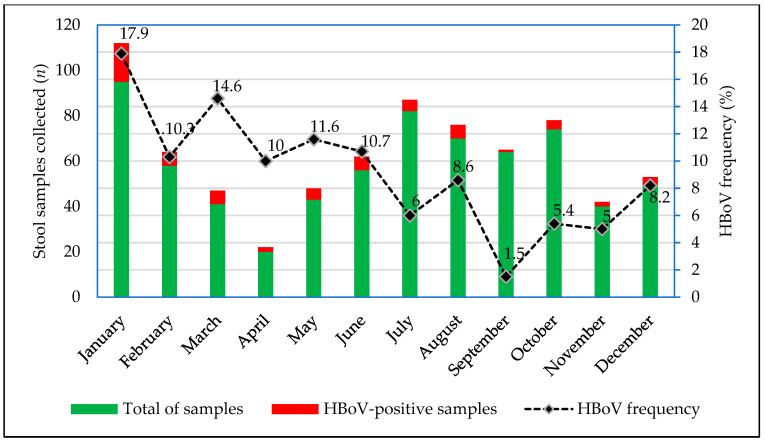
Monthly distribution of HBoV frequency among patients with AG in Northern Brazil (2017–2022).

Between 2017 and 2022, HBoV DNA was detected in 64 out of 692 patients (9.2%). The detection rate ranged according to the year: 12.6% (26/206) in 2017; 8.9% (13/146) in 2018; 17.2% (20/116) in 2019; 6.7% (1/15) in 2020; 0.8% (1/116) in 2021; and 3.2% (3/93) in 2022. Regarding monthly distribution, HBoV frequency fluctuated between 1.5% and 17.9%, with the highest rates (10% to 17.9%) detected in January to June, as demonstrated in Figure 1.

### 3.2. Epidemiological and Clinical Characteristics of HBoV Cases

HBoV positivity was slightly higher in males (57.8%) than in females (42.2%). Among the HBoV-positive cases, 70.3% were between 0 and 24 months old, and 15.6% were between 25 and 60 months old. Concerning clinical symptoms, 54.7% had fever; 67.2% vomiting; and 90.6% diarrhea (Table 1). No significant relationship was observed between HBoV-positive cases and clinical and epidemiological features.

### 3.3. Coinfection Between HBoV and Other Gastroenteric Viruses

Initially, the samples investigated had already undergone a screening process to identify other AG-causing viruses (RVA and NoV), since they are part of an epidemiological surveillance network. Single infections of HBoV were detected in 51.6% (33/64) of cases. The coinfection ratio between HBoV and other enteric viruses was reported in 48.4% (31/64) of positive samples. The most predominant HBoV coinfection was with RVA, detected in 31.2% (20/64) of cases, followed by NoV in 15.6% (10/64). A triple infection with all these agents was present in one specimen (1.6%, 1/64).

### 3.4. HBoV Genotypes

**Figure 2:**
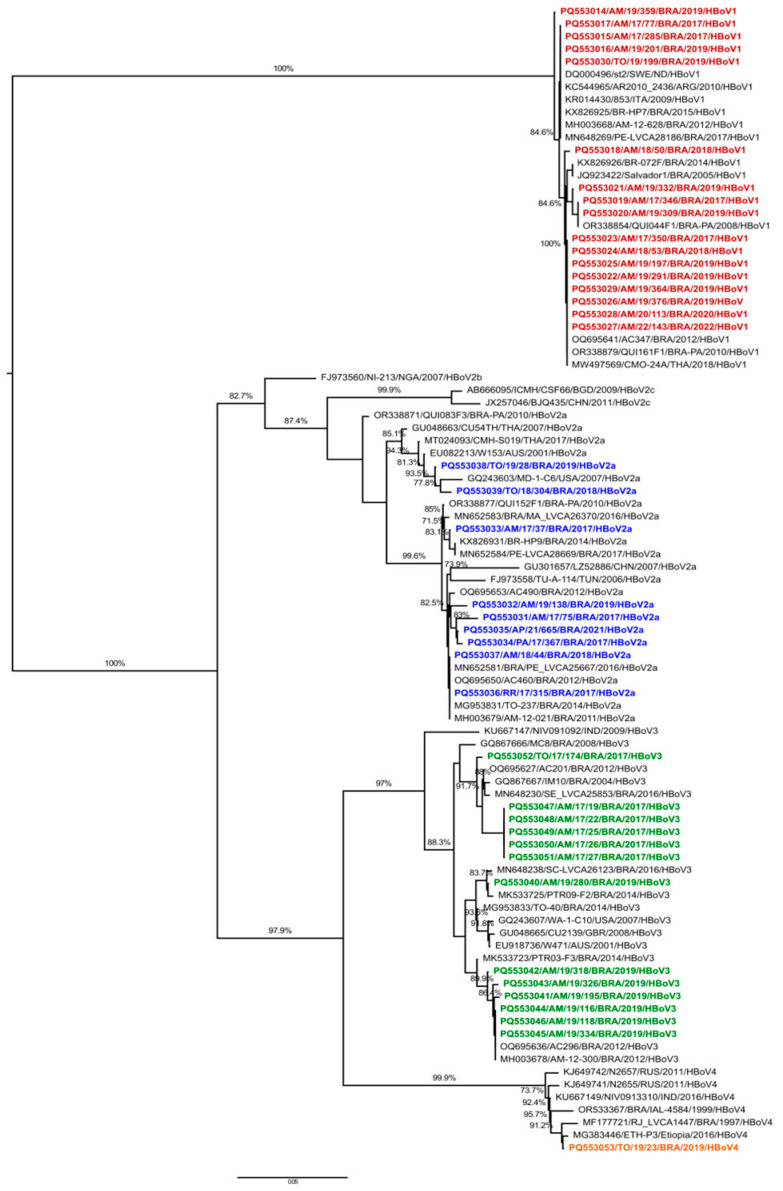
Phylogenetic tree analyses based on VP1/VP2 gene of HBoV strains from Northern Brazil (2017–2022). HBoV strains analyzed in the present study are marked in red (HBoV-1), blue (HBoV-2), green (HBoV-3), and orange (HBoV-4). Reference GenBank samples were included and accessed according to their reference numbers. The analysis was inferred by using the maximum likelihood method, including the GTR + Gamma + F nucleotide substitution model.

From the 64 detected positive HBoV DNA specimens, 40 (62.5%) were successfully genotyped. HBoV-1 was the most frequently detected species, responsible for 42.5% (17/40) of cases, followed by HBoV-3 (32.5%, 13/40), HBoV-2 (22.5%, 9/40), and HBoV-4 (2.5%, 1/40). Figure 2 shows the phylogenetic inference of the VP1/VP2 gene and demonstrates that HBoV-1 strains had high nucleotide (nt) similarities (99.3–100%) and amino acid (aa) (99.1–100%) similarities with strains from Europe, South America, and Asia. The HBoV-2 specimens clustered into lineage A with nt and aa similarities ranging from 93.4% to 99.8% and from 90.1% to 100%, respectively. The HBoV-3 samples were grouped into specimens from India, the EUA, Australia, the United Kingdom, and Brazil (nt similarity 96.1–100%, aa similarity 95.4–100%). A rare HBoV-4 genotype was detected in a child (aged 18 months) mono-infected with HBoV and presenting the classic triad of AG symptoms (diarrhea, vomiting, fever). This sample had a high similarity (nt 98.5–99.6%, aa 98.6–99.8%) with strains from Russia, India, Brazil, and Ethiopia.

## 4. Discussion

Studies on HBoV detection in fecal samples from individuals with AG have gained importance worldwide and aim to comprehend its role in this kind of infection [16,22,23]. In the present study, a detection rate of 9.2% was observed for HBoV among patients with AG symptoms in the North Region of Brazil. Similar data of positivity have been reported in Brazil and other settings with AG symptomatic patients. Kachooei et al. (2023) [24], in a study involving hospitalized children in Iran between 2021 and 2022, reported a positivity rate of 14%. In Brazil, a study published by Malta et al. (2020) [25] described an HBoV frequency of 12.4% in children up to two years of age. Trindade et al. (2023) [26] found a positivity rate of 10% in children with and without diarrhea symptoms in Acre during 2012.

Other studies reported lower frequencies. In Taiwan, HBoV was detected in 2.4% of samples from AG outbreaks that occurred between 2018 and 2022 [27]. In Thailand, the detection rate was 5.2% in pediatric patients with the same clinical condition (AG) during 2012–2018 [15]. In Brazil, Viana et al. (2024) [16] reported an HBoV positivity rate of 5.8% when analyzing retrospective AG samples from 1998 to 2005.

The HBoV positivity rate varies among studies, which can be explained by different influencing factors: (i) time and period of fecal sample collection; (ii) geographical disparity; and (iii) individual immunologic and nutritional aspects. These factors can directly contribute to the discrepancies related to detection rates [15,26].

Regarding the temporal distribution of HBoV cases, a decline in detection rates was observed during the COVID-19 pandemic (2019–2022). There was a significant overload on the public health surveillance system, with an almost exclusive focus on COVID-19 cases, which resulted in the underreporting of other diseases, such as gastroenteritis, as reported by Gutierrez et al. (2023) [28] when investigating the diversity and prevalence of RVA genotypes in children and adults presenting with AG symptoms in Brazil during the COVID-19 pandemic between 2020 and 2022.

Between 2017 and 2022, HBoV was detected in every month of the year, with the highest rates (10 to 17.9%) during the January to June period, corresponding to the Amazon winter. These results are consistent when compared to those of Viana et al. (2012) [29], who also identified monthly HBoV presence in historical fecal samples from Brazil and showed higher HBoV detection rates in autumn (April to June) and winter (July to September). It is important to note that the seasonality of HBoV is not well defined, highlighting the lack of consensus and significant correlation regarding specific seasonal patterns for this virus. However, several studies indicate an increase in HBoV cases during the rainy season, both in respiratory and AG infections, like other viral infections, as noted in the current analysis [25,30,31].

Regarding epidemiological features, HBoV was more frequent among male patients, accounting for 57.8% of cases. This trend aligns with the findings of Trindade et al. (2023) [26], who reported a higher incidence of HBoV among males (52.1%) in a study conducted in Acre, in the North Region of Brazil, in children with or without AG in 2012. Similarly, Kachooei et al. (2023) [24] described a higher HBoV incidence among males (64%). No significant association was found between HBoV infection and gender in these findings, suggesting a need to conduct more studies with larger sample sizes.

Concerning age groups, we found that the highest prevalence of HBoV positivity was observed in children aged 0 to 5 years, representing 85.9% of cases. These results agree with those of Viana et al. (2024) [16], who identified the highest frequency rate in this age group when analyzing historical HBoV samples collected between 1998 and 2005 in Brazil. Soares et al. (2019) [11] also observed that HBoV was found in 50.6% of acute AG cases among children under five years old in the North Region of Brazil from 2011 to 2012. A similar finding was reported by Chiu et al. (2024) [27] in Taiwan, where the highest infection rate occurred in individuals under three years old, with a prevalence of 46.6% during the period from 2018 to 2022. The higher incidence of HBoV cases in this age group (<5 years) may be due to the decline in maternal antibodies overlapping with the exposure to HBoV during infancy and early childhood [15].

With respect to clinical features in the present study, we observed the classic triad of AG symptoms in most patients infected with HBoV (90.6% presented diarrhea, 67.2% vomiting, and 54.7% fever). These findings are consistent with other studies worldwide. Rikhotso et al. (2020) [14] identified symptoms such as diarrhea (100%), fever (27%), vomiting (27%), dehydration (16%), respiratory tract infection (15%), and abdominal pain (15%) in outpatient children infected with HBoV from rural communities in South Africa. Sharif et al. (2020) [32] reported similar symptoms, with diarrhea (100%) and vomiting (57%) in HBoV-infected patients with gastroenteritis in Bangladesh from 2015 to 2019. Likewise, Soares et al. (2019) [11] observed fever (21.8%) and vomiting (17%) when analyzing the presence of HBoV in children under 10 years old in Northern Brazil between 2011 and 2012. Trindade et al. (2023) [26] also reported symptoms such as diarrhea, fever, and vomiting in 39.6%, 37.5%, and 16.7% of children infected with HBoV, with or without gastroenteritis symptoms. These symptoms are commonly observed in individuals with positive HBoV cases, often involving coinfection with other enteric viruses.

Coinfection between HBoV and other gastroenteric viruses was reported in 48.4% of cases, with HBoV + RVA being the most prevalent association detected in 31.2% of cases. Previous studies have also demonstrated coinfection between HBoV and other viral agents. Malta et al. (2020) [25] observed that only 20.9% of samples had HBoV as a single infection, while 23.7% were associated with NoV, 11% with RVA, and 2.7% with all three viruses. Soares et al. (2019) [11] also highlighted coinfection between HBoV and RVA in 50.0% (27/54) of the samples in their study on the molecular epidemiology of HBoV in children with gastroenteritis in the North Region of Brazil. However, it is important to note that, in the present study, mono-infection involving HBoV was detected in 51.6% of cases. Further studies are needed to assess the true role of HBoV in gastroenteritis cases, particularly analyses involving asymptomatic patients, and viral detection in respiratory specimens due to its potential role in respiratory infections, which may only be excreted through the gastrointestinal tract [31].

Although HBoV-1 is frequently identified in respiratory tract infections, it was the most common genotype identified in the fecal samples analyzed in this study (42.5%). Its association with gastroenteritis remains unclear, since this genotype can be shed in feces for months after infection [33]. However, several studies have reported its presence in diarrhea cases. In a study conducted by Chiu et al. (2024) [27] in Taiwan, HBoV-1 was also the most reported specie, responsible for 41.1% of AG cases. HBoV-1 was the most commonly detected genotype (79.7%) in historical fecal samples collected between 1998 and 2005 in Brazil [16]. Nevertheless, the present study did not include clinical data related to respiratory symptoms, which limits the ability to accurately determine the role of HBoV-1 as a causative agent of diarrhea in this population.

On the other hand, HBoV-2 and HBoV-3 are commonly identified in fecal samples from patients with AG, while their presence is rare in respiratory infections. The detection rate of HBoV-2 and HBoV-3 in this analysis was also significant, representing 22.5% and 32.5% of cases, respectively, corroborating with several studies pointing to the high incidence of these genotypes in AG cases [24,26,28,29].

It is important to note that a rare HBoV-4 genotype was detected in only one sample in the current analysis. HBoV-4 was first identified in 2010 during a multicenter study with fecal samples [4]. In Brazil, and in other countries around the world, its detection is uncommon. This species was first reported in Brazil in a child with symptoms of AG and respiratory tract infection with a soft tissue tumor in the submandibular region [34]. Recently, a report associated this genotype with gastroenteritis cases in a specimen collected in Brazil in 1999 [16].

The present study has some limitations: a lack of specific data on respiratory symptoms; the absence of additional tests for other enteric pathogens; no control group of healthy individuals (asymptomatic cases); a limited number of available samples for analysis. Despite these limitations, this study provided valuable information on the epidemiological and molecular profiles of HBoV, enhancing the current understanding of acute gastroenteritis infections in Northern Brazil. Future studies should consider including more detailed data on respiratory symptoms and increasing the sample size for more comprehensive and precise analysis.

## 5. Conclusions

The present study was conducted to evaluate the prevalence, clinical features, and genotypes distribution of HBoV in Northern Brazil. The data imply that HBoV was detected at a moderate frequency (10%) in AG cases; HBoV was most detected during the rainy season; children under 2 years of age were the most susceptible to HBoV contamination; and a rare HBoV-4 genotype was found. The study results underscore the importance of continuous monitoring and further research to better understand the seasonality, coinfection patterns, and genetic variability of HBoV. This information is crucial for developing effective public health policies aimed at mitigating the impact of gastroenteric infections caused by this emerging virus in the North Region of Brazil.

## Figures and Tables

**Table 1:** Epidemiological and clinical characteristics of HBoV-positive and -negative patients with acute gastroenteritis in Northern Brazil (2017–2022).

Features	HBoV Cases						
	Negative (*n* = 628)		Positive (*n* = 64)		Total (*n* = 692)		*p* Value
	N	(%)	N	(%)	N	(%)	
Gender							
Female	303	48.2	27	42.2	330	47.7	0.35
Male	325	51.8	37	57.8	362	52.2	
Age group (months)							
0–24	375	59.7	45	70.3	420	60.7	0.15
25–60	115	18.3	10	15.6	125	18.1	
>61	136	21.7	8	12.5	144	20.8	
NI *	2	0.3	1	1.6	3	0.4	
Clinical symptoms							
Diarrhea	570	90.8	58	90.6	628	90.7	0.62
Vomiting	405	64.5	43	67.2	448	64.7	0.75
Fever	323	51.4	35	54.7	358	51.7	0.47

* NI: no information.

## Data Availability

The datasets presented in this study can be found in online repositories. The names of the repository/repositories and accession numbers can be found in https://www.ncbi.nlm.nih.gov/genbank/, PQ553014-PQ553053 (accessed on 31 October 2024).
